# Neonatal interstitial lung disease in a girl with Jacobsen syndrome: a case report

**DOI:** 10.1186/s13256-022-03351-5

**Published:** 2022-03-24

**Authors:** Marit Lunde Dalen, Natalya Filipchuk Vigerust, Clara Hammarström, Henrik Holmstrøm, Jannicke Hanne Andresen

**Affiliations:** 1 Department of Neonatal Intensive Care, Division of Paediatric and Adolescent Medicine, Oslo University Hospital, Nydalen, Box 4956, 0424 Oslo, Norway; 2 Department of Medical Genetics, Oslo University Hospital, Oslo, Norway; 3 Department of Pathology, Rikshospitalet, Oslo University Hospital, Oslo, Norway; 4 Department of Paediatric Cardiology, Oslo University Hospital, Oslo, Norway; 5 Institute of Clinical Medicine, University of Oslo, Oslo, Norway; 6 Department of Neonatology, Oslo University Hospital, Oslo, Norway

**Keywords:** Neonate, Pulmonary hypertension, Thrombocytopenia, Immunodeficiency, Interstitial lung disease, Case report, Jacobsen syndrome

## Abstract

**Background:**

We report a case of the neonatal interstitial lung disease pulmonary interstitial glycogenosis in a girl with Jacobsen syndrome. While Jacobsen syndrome is caused by a deletion on the long arm of chromosome 11 and is genetically confirmed, pulmonary interstitial glycogenosis is of unknown etiology and is diagnosed by lung biopsy. Pulmonary interstitial glycogenosis has not previously been described in association with Jacobsen syndrome.

**Case presentation:**

A term newborn small for gestational age Caucasian girl presented with respiratory distress, pulmonary hypertension, congenital heart defects, immunodeficiency, and thrombocytopenia. She was diagnosed with Jacobsen syndrome, but also had pulmonary interstitial glycogenosis, which contributed to significant morbidity. There was striking clinical improvement after steroid treatment of the pulmonary interstitial glycogenosis.

**Conclusions:**

Interstitial lung disease should be considered as a differential diagnosis when respiratory distress and hypoxemia in the perinatal period worsens or persists despite standard treatment. Importantly, pulmonary interstitial glycogenosis may be treatable with corticosteroids. Whether there is a genetic link between pulmonary interstitial glycogenosis and Jacobsen syndrome is still unknown.

## Background

Interstitial lung disease (ILD) is a diverse group of rare conditions that cause impaired gas exchange and respiratory failure in the neonate. Neonatal ILD should be considered when respiratory distress and hypoxemia is out of proportion to gestational age and comorbidities, or worsens or persists despite adequate management. ILDs include developmental and growth abnormalities caused by genetic mutations, oligohydramnios, congenital diaphragmatic hernia, certain syndromes, and conditions of undefined etiology, including pulmonary interstitial glycogenosis (PIG) [1, 2]. Jacobsen Syndrome (JS) has a prevalence of 1 per 100.000 births, and is characterized by congenital heart disease, bleeding abnormalities, immunodeficiency, intellectual disability, specific facial features, and ocular findings [3–5]. To the best of our knowledge, PIG has not previously been reported in association with JS.

## Case presentation

A female small for gestational age (SGA) infant was delivered at 40 weeks 5 days of gestation by caesarean section after failed induction and fetal distress. She was the second child of healthy, unrelated Caucasian parents, and the pregnancy had been uneventful. Apgar score was 9-10-10. She had a birth weight of 2320 g, length of 45 cm, and head circumference of 34 cm, and was < 1st percentile for weight and height, and 17th percentile for head circumference. She was transferred to the neonatal intensive care unit due to persistent respiratory distress and cyanosis, with tachypnea at a rate of 75–80 and pulse oximetry saturations in the low 80’s, increasing to 98% with supplemental oxygen and continuous positive airway pressure (CPAP). She was intubated due to respiratory failure at 28 hours of age, but showed further deterioration with high oxygen demand (96%) on conventional ventilator, thus high-frequency oscillation ventilation (HFOV) was commenced. The initial chest x-ray showed bilaterally slightly reduced aeration with reticular opacities. Follow-up x-rays revealed atelectases and diffuse opacities. She had signs of circulatory failure with pale skin, hypotension and low urinary output. Echocardiography revealed persistent pulmonary hypertension with exclusively right-to-left shunt through the arterial duct, and inhaled nitric oxide (iNO) was initiated at 20 ppm. She received an intravenous (i.v.) fluid bolus, dopamine, and standard antibiotic treatment. The initial blood cultures were negative, and C-reactive protein was 18 (mg/L, normal < 4). Cranial ultrasonography was normal.

**Fig. 1:**
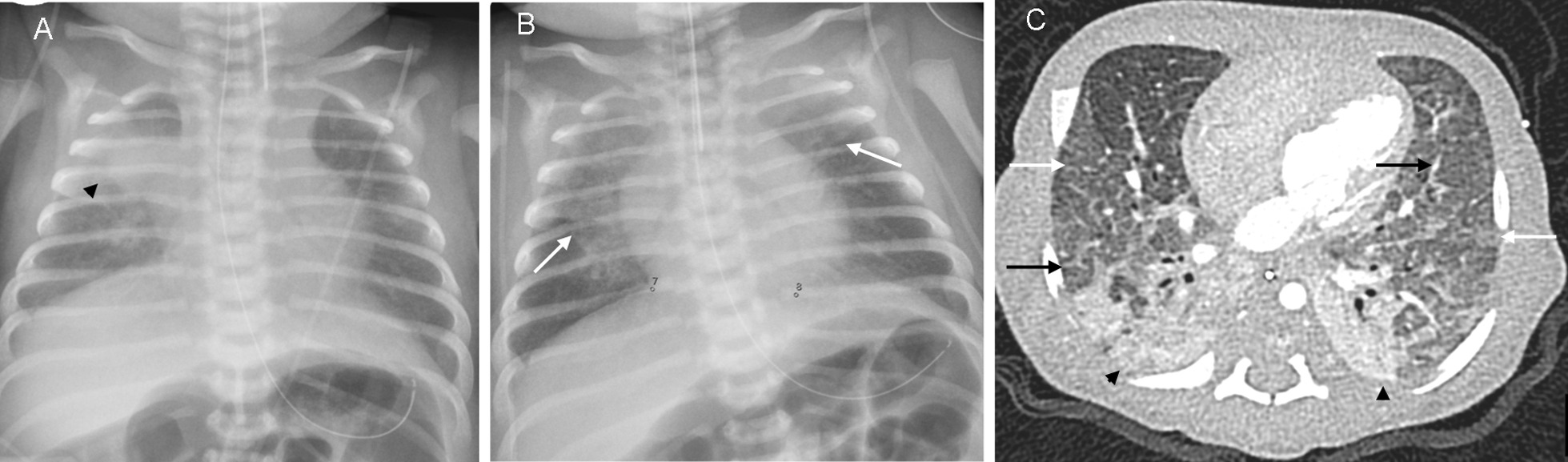
**A**–**C** Radiographs of the lungs acquired with a few hours interval on DOL 9 show: **A**, **B** intermittent atelectasis (black arrowhead) of the right upper lobe and central diffuse opacifications bilaterally (white arrows). A chest computed tomography (CT) at DOL 10 revealed: **C** Substantial atelectasis in the dorsal segments of the lungs (black arrowheads) and signs of thickened interlobular septae (black arrows) and scattered ground-glass opacifications (white arrows)

**Fig. 2:**
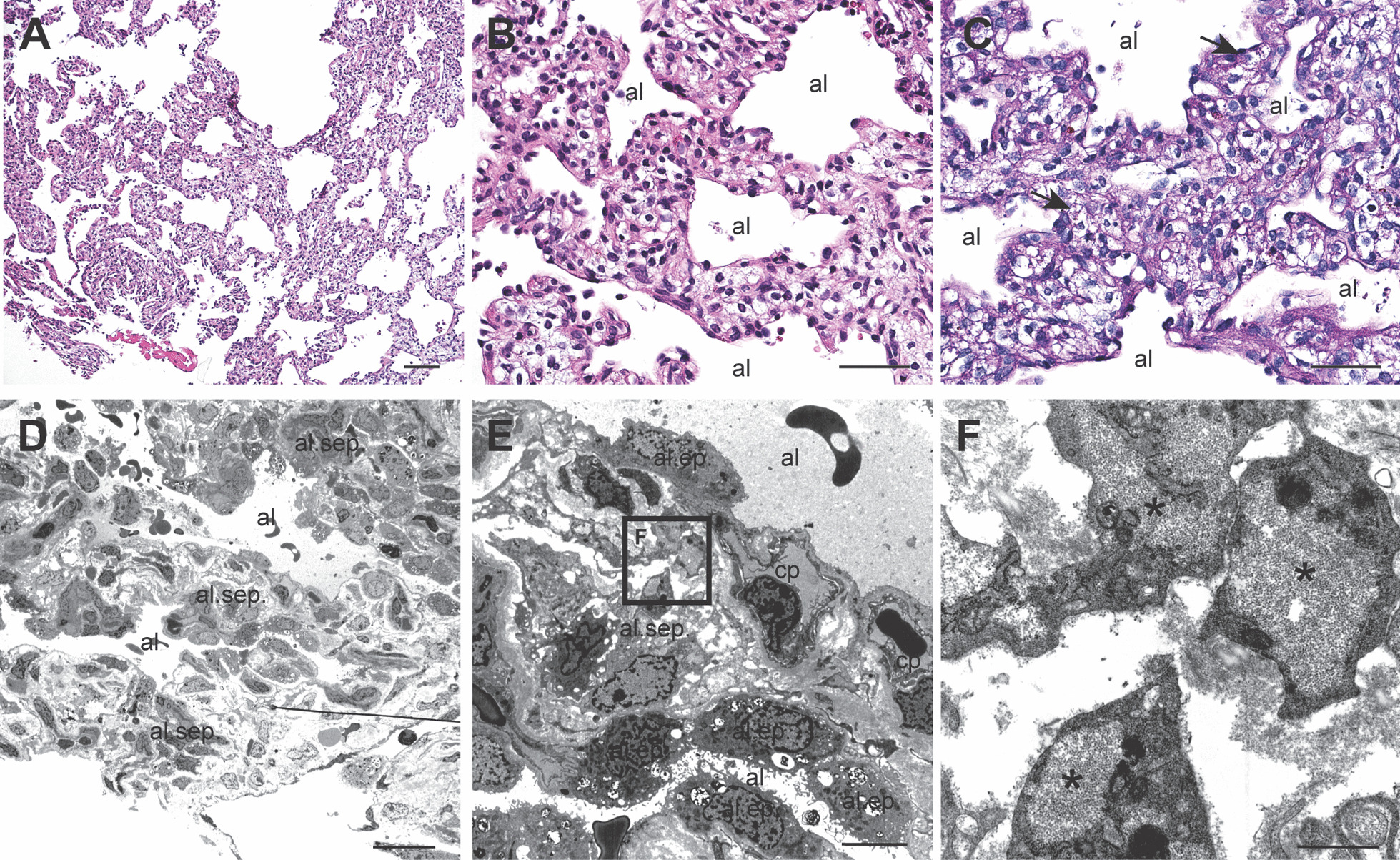
**A**, **B** Hematoxylin–eosin–saffron (HES) stain from lung wedge biopsy shows thickened alveolar septae and alveoli with reduced septation. **B** Increased amount of mesenchymal interstitial cells with clear cytoplasm in alveolar septae. **C** Periodic acid–Schiff (PAS) stain highlights glycogen granules (arrows) in the interstitial cells. **D**–**F** Transmission electron microscopy shows thickened alveolar septae (al.sep). The capillaries (cp) show normal distribution near the alveolar lumen (al). The alveolar epithelial cells (al.ep) contain normal amounts of surfactant. The interstitial cells in the alveolar septae contain increased amounts of glycogen granules (asterisk). Original magnification ×100 (**A**), ×400 (**B**, **C**). Scale bar: 100 mm (**A**), 50 mm (**B**, **C**), 20 µm (**D**), 5 µm (**E**), and 1 µm (**F**)

She was transferred to a tertiary hospital on the second day of life (DOL). Echocardiography confirmed significant pulmonary hypertension as well as a small ductus arteriosus, bicuspid aortic valve, perimembranous ventricular septum defect and atrial septum defect. The septal defects were considered insignificant, and the aortic valve had normal function. She was hypotensive and received circulatory support with dopamine and/or hydrocortisone until DOL 15. She received invasive respiratory support, mainly HFOV, until DOL 30, and iNO until DOL 35. Although there was adequate ventilation, she displayed significant oxygenation difficulties and iNO dependency, and had a failed extubation due to atelectases after 3 days of CPAP. The recurring atelectases and prolonged and unexpectedly severe respiratory failure warranted suspicion of a neonatal ILD. Analyses of genes related to surfactant deficiency and congenital alveolar capillary dysplasia were normal: ATP-binding cassette sub-family A member 3 (*ABCA3*), surfactant pulmonary-associated protein C and B (*SFTPC* and *SFTPB*, respectively) and forkhead box protein F1 (*FOXF1*). Radiologic investigations, including a chest computed tomography (CT) at DOL 10, revealed intermittent atelectasis, ground-glass opacifications, and signs of thickened interlobular septae (Fig. 1). A lung wedge biopsy at DOL 22 showed delayed maturation and relatively pronounced interstitial glycogenosis, consistent with PIG (Fig. 2). Four days prior to the biopsy she had suffered severe clinical deterioration and was given a single high dose of dexamethasone (750 µg), which had a striking clinical effect. After the diagnosis of PIG was confirmed, oral corticosteroid treatment was recommenced similar to the regimen described by Canakis *et al*. [6], but starting with dexamethasone of 500 µg/kg/day. She showed further rapid clinical improvement, and was extubated to CPAP 2 days later, weaned off iNO during the next 5 days, and without respiratory support from DOL 36.

There was significant thrombocytopenia from birth, with a platelet count of 47 (× 10^9^). Hemoglobin was 14.8 (g/dL) and white blood cell count was 8.9 (× 10^9^). She received two platelet transfusions, but still displayed sustained bleeding tendency, especially from mucous membranes. International normalized ratio (INR) was 1.5, and activated partial thromboplastin time (APTT) was 98 seconds. Abdominal ultrasonography revealed progressive splenomegaly. There were no signs of thrombi in the abdomen or neck, nor evidence of congenital viral infection as tests were negative for enterovirus, adenovirus, Parvo B19, cytomegalovirus and herpes simplex virus.

She developed clinical septicemia twice, first culture negative at DOL 12, then culture positive (*Staphylococcus aureus*) following the lung biopsy at DOL 22, both treated with broad-spectrum antibiotics. During the latter septicemia, she was hemodynamically unstable and received circulatory support (epinephrine, milrinone and sildenafil). Immunophenotyping confirmed a combined immunodeficiency, with reduced counts of B-cells and CD4+ T-cells. Although there was a normal relative number of naïve CD4+ T-cells, the portion of recent thymic emigrants (RTE) was reduced, which is consistent with reduced thymic output.

Repeated cranial ultrasonographies were normal, as were standard audiometry, ophthalmologic exam, thyroid stimulation hormone, and thyroxine. A comprehensive neurological assessment was not performed, but general clinical examination showed limb contractures, sparse spontaneous movements and facial mimics, with only brief eye contact and head lag. She was fully enteral fed via a nasogastric feeding tube by DOL 27, and had bottle training before discharge to the local hospital at PMA 46 + 4 weeks. Upon discharge, her weight was 2929 g, head circumference was 34.8 cm, and length was 50 cm (< first, second, and third centiles, respectively). She received sildenafil and dexamethasone, as well as prophylaxes of trimethoprim/sulfamethoxazole and fluconazole, and i.v. immunoglobin as needed. Pulsed treatment with methylprednisolone (monthly 3-day courses of 10 mg/kg/day) was continued until 4 months of age.

**Fig. 3:**
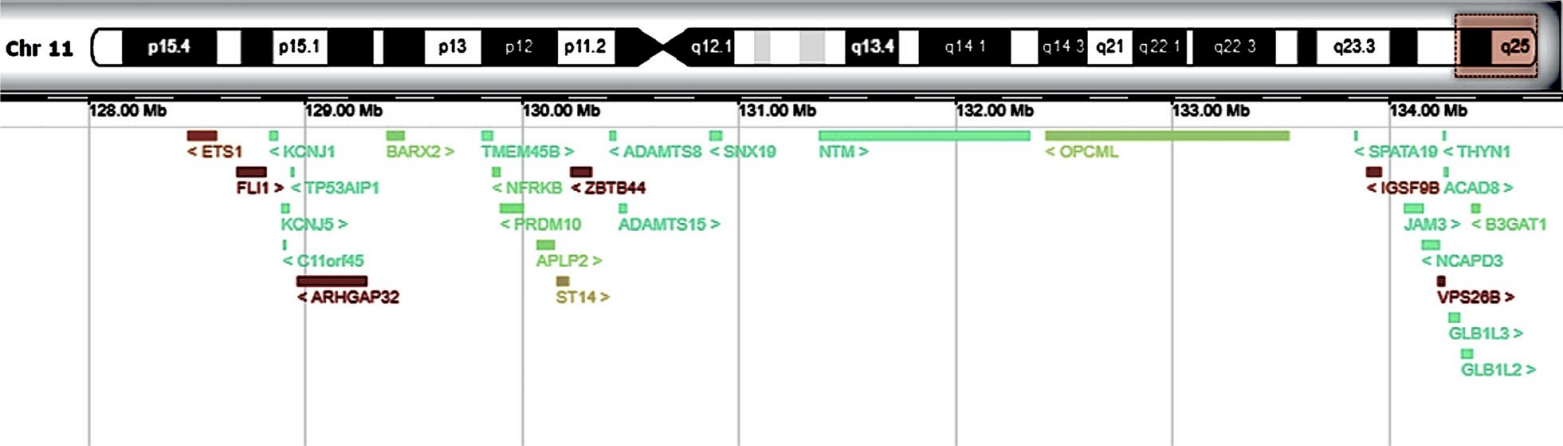
Graphic representation of the deleted region on chromosome 11, (arr [GRCh37] 11q24q25(127434377_134927114)x1), including the protein coding genes (from Decipher, https://decipher.sanger.ac.uk)

The diagnosis of JS was established by array-based comparative genomic hybridization showing a deletion of 7.5 Mb on chromosome 11 (Fig. 3): arr[GRCh37] 11q24q25(127434377_134927114)x1. This finding yielded a partial explanation of the phenotype, namely the cardiac defects, immunodeficiency, thrombocytopenia, and bleeding tendency, but not the ILD.

## Discussion and conclusions

PIG is a rare, idiopathic interstitial lung disease that usually manifests in the neonatal period as refractory respiratory distress with tachypnea and persistent hypoxemia. CT findings in isolated PIG are unspecific, but include ground-glass opacities, septal thickening, and cystic regions with diffuse or patchy distribution [2]. The diagnosis is confirmed by lung biopsy, revealing poorly differentiated mesenchymal cells that contain accumulated cytoplasmic glycogen, known as glycogenosis. Further characteristics of isolated PIG include diffuse interstitial thickening, normal development of capillaries and alveoli, and no signs of inflammation or infection [6–9]. However, PIG may be associated with other congenital lung disorders, pulmonary hypertension, congenital heart disease, mucopolysaccharidosis, Noonan syndrome, and trisomy 21 [8, 10–12]. It is not known whether PIG precedes or follows structural developmental defects, or if the affected cells contribute to, or are markers of, abnormal development. It has been suggested that PIG represents an underlying lung development disorder, a “pulmonary dysmaturity syndrome,” in the absence of inflammatory or infectious causes [6, 7, 10]. Although PIG has occasionally been described in patients with both chromosomal abnormalities and syndromes due to single-gene mutations, and abnormal stem cell differentiation has been reported in monozygotic twins with PIG [13], no candidate genes have been proposed. Thus, a potential link between the genes that play important roles in antenatal lung development and this diagnosis remains unrevealed.

Corticosteroid treatment may have a striking clinical effect in PIG [6] with complete resolution of changes confirmed by lung biopsy [14]. Since there is no inflammation, the postulated mechanism is promotion of tissue maturation, possibly through acceleration of lipofibroblast apoptosis [14]. Isolated PIG is believed to carry a favorable prognosis [6, 7, 12]. However, only two of nine asymptomatic infants diagnosed with PIG had normal lung function tests after 12 years, and abnormal CT findings were still present [15]. The prognosis is less favorable when PIG is associated with other conditions, such as congenital heart disease, pulmonary hypertension, lung lesions or genetic syndromes [8, 11].

Our patient was diagnosed with Jacobsen syndrome caused by the terminal deletion of the long arm of chromosome 11 (11q24q25). Typically, the deletion in patients with JS varies in size between 5 and 20 Mb and contains approximately 342 functional genes. Depending on the size and breakpoints of the deletion, the phenotype will vary [7]. About 85% of cases are *de novo* deletions, while 15% are the result of a balanced translocation or another chromosomal structural rearrangement (ring chromosome or a pericentric inversion) in one of the parents [3–5].

Historically, about 20% of children with JS have died during the first 2 years of life, most often due to congenital heart defects [4] which are found in more than 50% of patients, mainly ventricular septum defects and left-sided heart lesions [16]. Neonatal thrombocytopathy and transient thrombocytopenia is seen in 90%, and is known as Paris–Trousseau syndrome [5, 17]. Bleeding severity greater than predicted by platelet count is caused by dysfunctional giant alpha granules that fail to release contents in response to thrombin. This platelet dysfunction usually persists [5]. Antibody deficiency is common, and T-cell defects may also be observed. Thus, the immunodeficiency in JS is considered a primary combined immunodeficiency in need of monitoring and prophylaxes. While respiratory tract infections and chronic diarrhea are common, severe bacterial infections with fatal outcome may also occur [18]. Further characteristics include SGA, hypotonia, postnatal growth retardation, delayed psychomotor development, and mild to severe degree of intellectual disability. Brain anomalies have been reported [19], as well as ocular findings including anomalies of extraocular muscles, amblyopia, microcornea, opticus atrophy, macular hypoplasia, and chorioretinal coloboma [20]. Typical facial features include hypertelorism, telecanthus, ptosis, downslanting palpebral fissures, epicanthus, broad nasal bridge and short nose, v-shaped mouth, and small low-set posteriorly rotated ears [4, 5]. To our knowledge, interstitial lung disease has not been described as part of the JS phenotype so far.

This case report describes two rare diseases occurring in association: PIG and Jacobsen syndrome. Although this could be coincidental, we find the similarity to another recent case report highly interesting: a near-term infant with JS required respiratory support for 54 days and supplemental oxygen until DOL 67 [21]. It remains a subject of speculation whether this represented an interstitial lung disease such as PIG that could potentially have benefited from early corticosteroid therapy. The gene region (Fig. 3) affected in JS contains *ADAMTS8* (a disintegrin-like and metalloproteinase with thrombospondin motif 8). *ADAMTS8* is less described than the other members of the *ADAMTS* family, yet it is highly expressed in heart and lung tissue. It belongs to a group of enzymes with ability to cleave proteoglycans, thus regulating the structure and function of extracellular proteins in blood and extracellular matrix. The proteoglycanases can regulate cell signaling, proliferation, migration, and apoptosis through their interaction with extracellular matrix proteins, growth factors and chemokines [22].

Consistent with JS, our patient had congenital heart defects, abnormal thrombocyte function and a combined immunodeficiency. However, significant morbidity was caused by prolonged and unexpectedly severe respiratory failure and pulmonary hypertension that was out of proportion to both gestational age and type of cardiac defect. She was finally diagnosed with PIG and JS, two rare conditions not previously reported in association. We maintain that ILD should be considered a differential diagnosis when respiratory distress and hypoxemia in the perinatal period worsens or persists despite standard treatment. Importantly, PIG may be treatable with corticosteroids and should be considered when ILD is suspected in the neonate. It remains a subject of speculation whether there is a genetic link between PIG and Jacobsen syndrome.

## Data Availability

Data sharing is not applicable for this article as no datasets were generated or analyzed for the case report.

## Abbreviations

aCGH: Array-based comparative genomic hybridization
CPAP: Continuous positive airway pressure
CT: Computed tomography
DOL: Day of life
HES: Hematoxylin–eosin–saffron
HFOV: High-frequency oscillation ventilation
ILD: Interstitial lung disease
iNO: Inhaled nitric oxide
JS: Jacobsen syndrome
PAS: Periodic acid–Schiff
PIG: Pulmonary interstitial glycogenosis
